# Assesment of Generative AI abilities to diagnose and propose treatment in comparison with psychiatrists from Poland and Tunisa

**DOI:** 10.1192/j.eurpsy.2024.1145

**Published:** 2024-08-27

**Authors:** M. Sinica, A. Malec, H. Sghaier, P. Kalkowski

**Affiliations:** ^1^Psychiatry, Milickie Centrum Medyczne, Wrocław; ^2^Centralny Ośrodek Badań i Karier, Naczelna Izba Lekarska, Warszawa; ^3^Specialty Training Section, Polish Psychiatric Association, Warsaw, Poland; ^4^Psychiatry, Hedi Chaker university hospital, sfax, Tunisia; ^5^Centralny Ośrodek Badań i Karier, Naczelna Izba Lekarska, Warsaw, Poland

## Abstract

**Introduction:**

Increasing popularity of Generative AI systems such as GPT provides us with new dilemmas concerning the future of diagnosis and novel tools to improve daily psychiatrits’s work.

**Objectives:**

The aim of the study was to assess the abilities of generative AI to diagnose and propose treatment in comparison with real psychiatrists and performing a Turing test.

**Methods:**

We examined the ability to diagnose and propose treatment of various Generative AI versions (CHatGPT/CHATGPTpro etc.) and then compare the results with 10 clinicians performing the same task. Then a group of 10 psychiatry specialists not involved in the first evaluation assessed wether the diagnose and treatment were established by Generative AI or a clinician.

**Results:**

The reults showed that the generative AI systems were able to provide valid diagnosis in most of the cases with favour to newer and most proficient version of CHATGPT. Proposed treatment results were less accurate. The comparison between human and AI group was hard to accurately acces, with tendency to favouring psychiatrists group assesment as the right decision.

There is huge need to further explore the possibilites and limitations of Generative AI use in psychiatry.

**Conclusions:**

There is huge need to further explore the possibilites and limitations of Generative AI use in psychiatry.

**Disclosure of Interest:**

None Declared

